# The Effect of Endurance Exercise on Semen Quality in Male Athletes: A Systematic Review

**DOI:** 10.1186/s40798-024-00739-z

**Published:** 2024-06-11

**Authors:** Alex Aerts, Annelien Temmerman, Arne Vanhie, Dirk Vanderschueren, Leen Antonio

**Affiliations:** 1 Faculty of Medicine, Katholieke Universiteit Leuven, Leuven, Belgium; 2 Department of Internal Medicine, University Hospitals Leuven, Leuven, Belgium; 3 Leuven University Fertility Centre, University Hospitals Leuven, Leuven, Belgium; 4 Clinical and Experimental Endocrinology, Department of Chronic Diseases and Metabolism, KU Leuven, Leuven, Belgium; 5 Department of Endocrinology, University Hospitals Leuven, Leuven, Belgium

**Keywords:** Male reproduction, Semen quality, Endurance exercise

## Abstract

**Background:**

Endurance exercise has the potential to affect reproductive function, with amenorrhea in female athletes. However, most studies focus on women. Evidence on the association between endurance exercise and male fertility is limited.

**Objective:**

To synthesise existing literature on exercise-induced alterations in semen parameters and to assess the clinical impact on male fertility.

**Methods:**

Studies reporting on the association between semen parameters and endurance exercise in healthy men were eligible. Men attending fertility clinics were excluded. We searched MEDLINE (PubMed), Embase, SPORTDiscus, Cochrane Central Register of Controlled Trials (CENTRAL), ClinicalTrials.gov and International Clinical Trials Registry Platform (ICTRP) from their inception to May 28th 2022. JBI Critical Appraisal Tool was used to assess the potential risk of bias.

**Results:**

Thirteen studies met inclusion criteria, reporting on 280 subjects. Eight articles reported on endurance runners, three on cyclists and four on triathletes. Four studies did not find any statistically significant sperm alterations. Five reported significant changes in semen parameters, but these were not clinically relevant, as semen parameters remained well above World Health Organisation (WHO) thresholds. Four articles reported a decrease in semen quality with potential clinical consequences as they found a reduced number of sperm cells exhibiting normal morphology in cyclists and triathletes and a greater amount of DNA fragmentation in triathletes.

**Conclusion:**

Endurance exercise can have a negative effect on semen quality, although rarely with a clinically relevant impact on male fertility. Evidence is however limited, with poor quality of the included studies.

*Registration*: PROSPERO International prospective register of systematic reviews (CRD42022336753).

**Supplementary Information:**

The online version contains supplementary material available at 10.1186/s40798-024-00739-z.

## Key Points

- Results suggest that endurance exercise can decrease semen quality, although without a clinically relevant impact on male fertility potential.
- The available evidence is limited and lacks comparability due to poor methodology. High quality studies are necessary to further assess the relationship between endurance exercise and semen quality.

**Supplementary Information:**

The online version contains supplementary material available at 10.1186/s40798-024-00739-z.

## Background

Practicing moderate physical activity on a regular basis is associated with multiple health benefits. There is strong evidence that exercise has a preventive effect on pathogenesis and also leads to better symptom control in various somatic and psychiatric disorders [1]. Nonetheless, prolonged exercise may induce a condition called “overtraining”, which can be harmful to numerous physiological pathways [2]. Research mainly focusses on female athletes because they present with clinically visible symptoms such as amenorrhea. Amenorrhea is a component of the “Female Athlete triad” together with low energy availability (LEA) and decreased bone mineral density [3, 4]. Unfavourable reproductive health consequences in male athletes are less studied due to the absence of clinical signs and symptoms, although there is ongoing interest in the effect of endurance exercise on semen quality [4, 5].

So far, the literature has been ambiguous whether exercise affects spermatogenesis or not. Several theories on a possible association have been formulated. First, since spermatogenesis depends on testicular testosterone production, many authors focus on exercise-induced hormonal changes in male athletes [6]. In 2014 “Relative Energy Deficiency in Sport” (REDs) was introduced [7]. This is a comprehensive term for the condition previously known as “Female Athlete Triad”, as it now also includes low testosterone and fertility problems in male athletes. LEA is the underlying cause of REDs and can be defined as a mismatch between energy intake and expenditure, thereby leaving insufficient energy for metabolic pathways and disrupting the normal function of the hypothalamic-pituitary–gonadal (HPG) axis [7]. LEA can lead to low gonadotropin concentrations, low testosterone and subsequent reproductive problems in male athletes [8]. Furthermore, there are other consequences of endurance exercise that can negatively influence spermatogenesis. The increase in body temperature and wearing tight clothing during exercise may elevate scrotal temperature leading to impaired spermatogenesis [9–12]. Moreover, strenuous exercise causes excessive formation of reactive oxygen species (ROS), which can affect sperm DNA [13–17]. Also, there are sport-specific factors, such as sitting on a bicycle seat leading to mechanical compression of the testis, epididymis and vas deferens. This may induce testicular microtrauma and reduce testicular blood flow [18] and impair the secretory function of the prostate gland, which normally enhances the motility of spermatozoa [19].

Spermatogenesis is a multifactorial process, lasting approximately 70 days, in which germ cells undergo mitotic cell division, meiosis and spermiogenesis to become mature spermatozoa [20, 21]. This process is hormonally controlled by the HPG axis. By secreting gonadotropin-releasing hormone (GnRH), the hypothalamus stimulates the pituitary gland to release gonadotropins. Follicle stimulating-hormone (FSH) stimulates Sertoli cells to support spermatogenesis. Luteinizing hormone (LH) activates Leydig cells to produce testosterone, which is necessary for normal spermatogenesis and maturation of spermatozoa [22]. Semen analysis is a delicate procedure [23]. It is crucial to process and analyse semen samples in a standardized way, with clear instructions about abstinence time [24, 25]. Moreover, to account for individual variability in semen parameters, two consecutive semen samples should be examined [26].

In this systematic review, we aim to provide a comprehensive synthesis of the existing literature investigating exercise-induced alterations in semen parameters.

## Methods

We wrote this systematic review guided by the Preferred Reporting Items for Systematic Reviews and Meta-Analyses (PRISMA) 2020 checklist in order to report a transparent and complete review [27]. We registered the protocol for this systematic review before data extraction on PROSPERO International prospective register of systematic reviews (CRD42022336753). Our systematic review was approved by the KU Leuven ethics committee (MP018647).

### Search Strategy

The literature search was conducted according to the PRISMA-S checklist [28]. A comprehensive search strategy was developed by two authors (AA and LA), assisted by a staff member of the 2Bergen library of the Biomedical Sciences Group at KU Leuven. Our search strings consisted of MeSH terms, Emtree terms, keywords and free text. We searched four databases: MEDLINE (PubMed), Embase, SPORTDiscus and Cochrane Central Register of Controlled Trials (CENTRAL). Additionally, we explored two registers: ClinicalTrials.gov and International Clinical Trials Registry Platform (ICTRP). These sources were consulted from inception to May 28th 2022. For each platform, we modified the search string to implement database-specific filters and search terms. The complete search strategy is described in Supplementary Table S1.

### Eligibility Criteria

There were no limitations regarding publication date. We limited our search to English, Dutch or French articles but only retrieved English records. Case reports, qualitative studies and animal studies were excluded.

Studies that reported on the relationship between semen characteristics and endurance exercise were eligible. The subjects were required to meet the following criteria: men without chronic illnesses or reproductive problems, and detailed reporting of practicing endurance exercise. Therefore, men attending fertility clinics were excluded to avoid bias secondary to disturbed baseline sperm characteristics. Interventions had to be expressed in quantitative numbers, for example training volume or metabolic equivalent of task (MET). Outcomes were defined as semen parameters (total sperm count, sperm concentration, motility, morphology, total motile sperm count, DNA fragmentation).

### Selection Process

**Fig. 1:**
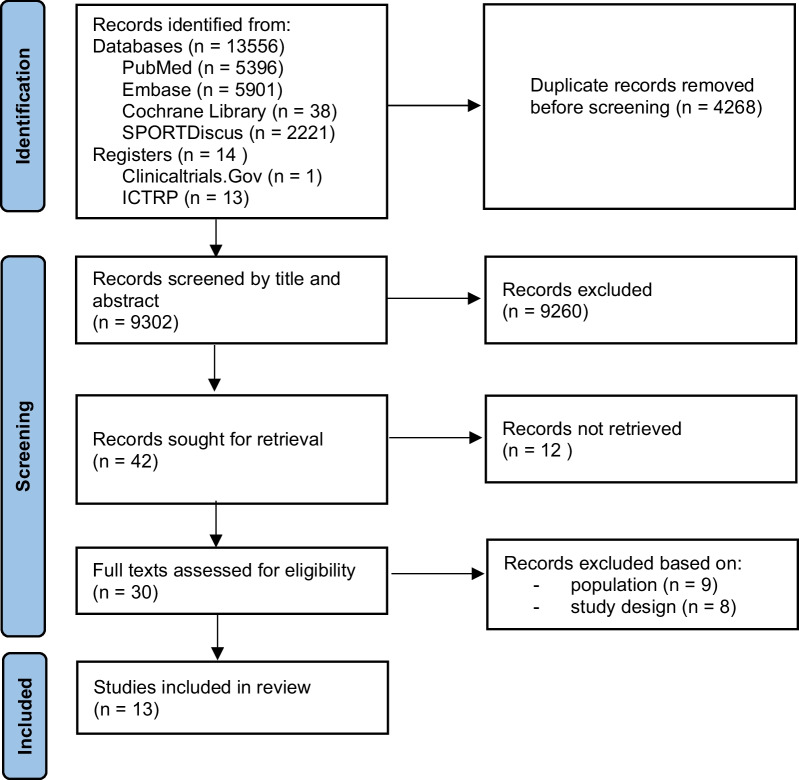
PRISMA 2020 flow diagram of study selection

After performing the search, duplicates were manually removed in EndNote20. To avoid reporting bias, two authors (AA and TA) independently screened all articles for eligibility at title/abstract stage and at full-text stage, using the systemic review software application Rayyan. For titles and abstracts that seemed relevant, full texts were retrieved and evaluated against the inclusion and exclusion criteria. Furthermore, we screened the reference lists of retrieved studies to find other relevant reports. Discrepancies were resolved by discussion. The search process was presented by the PRISMA flow diagram, along with reasons for exclusion (Fig. 1).

### Data Collection Process, Synthesis Methods and Risk of Bias Assessment

The data from all eligible studies were collected by one author (AA). The following information was extracted: title, author, country of origin, year of publication, study design, participants, interventions, methods, outcome measures and summary of results. The data extraction was carried out using a pre-defined form in Microsoft Excel. A complete description of the extracted data is reported in Supplementary Tables S2, S3 and S4. For the syntheses, reports were categorized by sport (running, cycling, triathlon) and study type (observational versus longitudinal). One reviewer (AA) assessed methodological quality and the potential risk of bias using the JBI Critical Appraisal Tool (Supplementary Table S5) [29].

## Results

### Study Selection

Our search yielded a total of 13,570 records, published between 1915 and 2022. After deduplication, 9302 unique records were left. 9260 articles were excluded by title and abstract screening. For 12 out of the 42 remaining records, we could not retrieve the full text. The full texts of 30 reports were assessed for eligibility of which 17 studies were excluded based on population or study type. Eventually, 13 studies were included.

### Study Characteristics

Eight articles reported on endurance running [19, 30–36], three on cycling [10, 19, 37], and four on triathlon [19, 38–40]. From the 13 eligible articles, seven (54%) had a cross-sectional design [10, 30, 32, 34, 38–40] and six (46%) were longitudinal [19, 31, 33, 35–37]. In the included articles, different guidelines and criteria for semen analysis were used (Supplementary Table S6). Two studies followed the criteria described by Bremner et al. in 1981 [31, 36], one the Kruger’s strict criteria of 1986 [35], two the Kruger’s strict criteria of 1995 [38, 40], two the WHO 2nd edition of 1987 [32, 34], one the WHO 3th edition of 1992 [19], two the WHO 4th edition of 1999 [10, 38], one the WHO 5th edition of 2010 [39] and three did not specify criteria used for semen analysis [30, 33, 37]. Years of publication ranged from 1985 to 2018. The majority of the articles were published in the United States (n = 7). The country of origin of the remaining articles was Spain (n = 4) and South Africa (n = 2). The included reports identified a total of 280 subjects who underwent semen analysis. However, it is unclear whether two studies used identical subjects [38, 40]. Characteristics of the included articles are presented in Tables 1, 2 and 3.

**Table 1:** Characteristics of included studies investigating endurance running

Study	Study design	Participants Age (years)	Intervention	Methods	Outcomes
Ayers et al. (1985) USA [30]	Cross-sectional	Endurance runners (n = 20) (48–129 km–w) Sedentary controls (n = 10) Age: 26–42 yr	–	*Other assessments:* hormonal: yes TT, FT, LH, DHEA-S, E_2_, PRL body composition: yes energy balance: no *Sperm analysis criteria:* Unspecified *Number of semen samples:* 1	Sperm count Sperm morphology
Bagatell et al. (1990) USA [31]	Longitudinal	Endurance runners (n = 12) (> 64 km–w) Sedentary controls (n = 12) Age: 21–37 yr	–	*Other assessments:* hormonal: yes TT, FT, SHBG, FSH, LH, C body composition: yes energy balance: no *Sperm analysis criteria:* Bremner et al. (1981) *Number of semen samples:* 6 (1 at 2-week intervals for 12 weeks)	Sperm count Total spermatozoa per ejaculate Sperm motility (forward progressive) Sperm morphology (oval forms)
Arce et al. (1993) USA [32]	Cross-sectional	Endurance runners (n = 10) (109.2 ± 4.8 km–w) Sedentary controls (n = 10) (< 1 h–w) Resistance-trained weightlifters (n = 8) (> 2 h–x, > 4 x–w) Age: 18–35 yr	–	*Other assessments:* hormonal: yes TT, FT, LH, FSH, E_2_, PRL body composition: yes energy balance: no *Sperm analysis criteria:* WHO 2nd edition *Number of semen samples:* 2–5	Sperm volume Sperm density Total sperm count In vitro penetration of bovine cervical mucus Sperm motility (forward progressive, non-progressive, non-motile) Sperm morphology (normal, immature, round cells)
Roberts et al. (1993) USA [33]	Longitudinal	Endurance-trained men (n = 5) (running, swimming and cycling) (> 4 d–w) Age: 23–26 yr	Doubling weekly mileage at constant intensity (2 weeks)	*Other assessments:* hormonal: yes TT, C body composition: yes energy balance: yes *Sperm analysis criteria:* Unspecified *Number of semen samples:* 4 before overtraining (6–8-week intervals) 1 immediately after overtraining 1 after 3 months	Sperm concentration (before overtraining, immediately after, 3 months after overtraining) Sperm morphology and sperm motility
De Souza et al. (1994) USA [34]	Cross-sectional	High mileage endurance runners (n = 11) (108.0 ± 4.5 km–w) Moderate mileage runners (n = 9) (54.2 ± 3.7 km–w) Sedentary controls (n = 10) (< 1 h–w) Age: 18–35 yr	–	*Other assessments:* hormonal: yes TT, FT, LH, FSH, PRL, ACTH, C, DHEA-SO_4_, T_3_, FT_3_, T_4_, FT_4_ body composition: yes energy balance: yes *Sperm analysis criteria:* WHO 2nd edition *Number of semen samples:* 2–5	Sperm volume Sperm density Sperm count Normal motile count Motile count Sperm penetration of cervical mucus Sperm motility (forward progressive, non-progressive, non-motile) Sperm morphology (normal, large, small, amorphous, immature, round cells)
Jensen et al. (1995) South Africa [35]	Longitudinal	Endurance runners (n = 24) Age: 25–54 yr	High training months: 60–160 km–w Low training months: < 55 km–w (over a 12 months period)	*Other assessments:* hormonal: yes TT, LH, FSH, PRL, E_2_, P body composition: yes energy balance: no *Sperm analysis criteria:* Kruger's strict criteria (1986) for morphology Number of semen samples: 1 in each of the following months: December, January, February, April, May, August, November	Sperm volume Sperm count Sperm morphology Sperm motility
Lucía et al. (1996) Spain [19]	Longitudinal & cross-sectional	Professional cyclists (n = 12) (884.46 ± 44.7 km–w) Elite triathletes (n = 9) running: 54.36 ± 7.2 km–w swimming: 14.36 ± 5.8 km–w cycling: 316.16 ± 79.0 km–w Marathon runners (n = 10) (94.26 ± 27.1 km–w) Sedentary controls (n = 9) Age: 22–38 yr	Follow-up during one sports season: precompetition competition resting period (2 weeks)	*Other assessments:* hormonal: yes TT, FT, FSH, LH, C body composition: yes energy balance: no *Sperm analysis criteria:* WHO 3rd edition *Number of semen samples:* 1 in precompetition 1 in competition 1 after resting period	Semen morphology, volume and density Sperm motility (competition, resting period) Note: absolute data were not mentioned but depicted in graphics
Hall et al. (1999) USA [36]	Longitudinal	Endurance runners (n = 8) (32–64 km–w) Sedentary controls (n = 8) Age: 19–37 yr	2 weeks normal training (NT) 2 weeks at 143% NT (IT1) 2 weeks at 186% NT (IT2) 2 weeks at 50% NT (RT)	*Other assessments:* hormonal: yes TT, FT, FSH, LH, PRL, C body composition: no energy balance: no *Sperm analysis criteria:* Bremner et al. (1981) *Number of semen samples:* 1 after 2 weeks NT 1 after 2 weeks IT1 1 after 2 weeks IT2 1 after 2 weeks RT	Sperm motility and morphology Sperm count Note: absolute data were not mentioned but depicted in graphics

TT: total testosterone; FT: free testosterone; LH: luteinizing hormone; DHEA-S: dehydroepiandrosterone-sulfate; E2: estradiol; PRL: prolactin; SHBG: sex hormone binding globulin; FSH: follicle stimulating hormone; C: cholesterol; ACTH: adrenocorticotropic hormone; DHEA-SO4: dehydroepiandrosterone-sulfate; T3: total triiodothyronine, FT3: free triiodothyronine; T4: thyroxine, FT4: free thyroxine; P: phosphate; NT: normal training; IT: interval training; RT: reduced training;

**Table 2:** Characteristics of included studies investigating cycling

Study	Study design	Participants Age (years)	Intervention	Methods	Outcomes
Griffith et al (1990) USA [37]	Longitudinal	Biathletes (n = 6): running: 40–65 km–w cycling: 85–190 km–w weightlifting: 2-3x–w Age: 22–44 yr	Double bicycling hours (2 weeks)	*Other assessments:* hormonal: yes TT body composition: yes energy balance: no *Sperm analysis criteria:* Unspecified *Number of semen samples:* 1 baseline 1 in follow-up	Sperm count (before overtraining, after overtraining)
Lucía et al (1996) Spain [19]	Longitudinal & cross-sectional	Professional cyclists (n = 12) (884.46 ± 44.7 km–w) Elite triathletes (n = 9) running: 54.36 ± 7.2 km–w swimming: 14.36 ± 5.8 km–w cycling: 316.16 ± 79.0 km–w Marathon runners (n = 10) (94.26 ± 27.1 km–w) Sedentary controls (n = 9) Age: 22–38 yr	Follow-up during one sports season: - precompetition - competition - resting period (2 weeks)	*Other assessments:* hormonal: yes TT, FT, FSH, LH, C body composition: yes energy balance: no *Sperm analysis criteria:* WHO 3rd edition *Number of semen samples:* 1 in pre-competition 1 in competition 1 after resting period	Semen morphology, volume and density Sperm motility (competition, resting period) Note: absolute data were not mentioned but depicted in graphics
Gebreegziabher et al (2004) South Africa [10]	Cross-sectional	Non-professional cyclists (n = 10) (> 40 min–d, > 3 d–w) Sedentary controls (n = 10) Age: 20–29 yr	–	*Other assessments:* hormonal: no body composition: yes energy balance: no *Sperm analysis criteria:* WHO 4th edition *Number of semen samples:* Unspecified	Sperm volume Sperm count Total sperm count Sperm motility (at time 0, after 2 h, after 4 h) Sperm morphology (normal, tapered, small acrosome, double head, immature forms, other forms) Sperm viability

TT: total testosterone; FT: free testosterone; FSH: follicle stimulating hormone; LH: luteinizing hormone; C: cholesterol

**Table 3:** Characteristics of included studies investigating triathletes

Study	Study design	Participants Age (years)	Intervention	Methods	Outcomes
Lucía et al (1996) Spain [19]	Longitudinal & cross-sectional	Professional cyclists (n = 12) (884.46 ± 44.7 km–w) Elite triathletes (n = 9) running: 54.36 ± 7.2 km–w swimming: 14.36 ± 5.8 km–w cycling: 316.16 ± 79.0 km–w Marathon runners (n = 10) (94.26 ± 27.1 km–w) Sedentary controls (n = 9) Age: 22–38 yr	Follow-up during one sports season: precompetition competition resting period (2 weeks)	*Other assessments:* hormonal: yes TT, FT, FSH, LH, C body composition: yes energy balance: no *Sperm analysis criteria:* WHO 3rd edition *Number of semen samples:* 1 in pre-competition 1 in competition 1 after resting period	Semen morphology, volume and density Sperm motility (competition, resting period) Note: absolute data were not mentioned but depicted in graphics
Vaamonde et al. (2009) Spain [38]	Cross-sectional	Physically active (n = 16) (> 1 h, 3x–w) (non-professional basketball, soccer, tennis, paddle ball) Water polo players (n = 14) (1.5 h, 5x–w) Elite triathletes (n = 15) running: 49.4 ± 7.4 km–w swimming: 11.3 ± 3.0 km–w cycling: 330.8 ± 56.0 km–w Age: 17–38 yr	–	*Other assessments:* hormonal: no body composition: yes energy balance: no *Sperm analysis criteria:* WHO 4th edition Kruger's strict criteria (1995) for morphology *Number of semen samples:* Unspecified	Sperm volume Sperm concentration Total sperm number Sperm morphology (normal forms) → Sperm motility^a^ (type "a", type "b", type "c", type "d")
Vaamonde et al. (2009) Spain [40]	Cross-sectional	Elite triathletes (n = 15) running: 49.42 ± 7.37 km–w swimming: 11.31 ± 3.05 km–w cycling: 330.77 ± 56.04 km–w Age: 29–38 yr	–	*Other assessments:* hormonal: no body composition: yes energy balance: no *Sperm analysis criteria:* Kruger's strict criteria (1995) for morphology *Number of semen samples:* Unspecified	Sperm morphology correlation (total weekly volume, cycling volume, running volume, swimming volume)
Vaamonde et al. (2018) Spain [39]	Cross-sectional	Elite triathletes (n = 12) running: ± 2.600 km–y swimming: ± 416 km–y cycling: ± 13.000 km–y Age: 24–30 yr	–	*Other assessments:* hormonal: yes TT, C body composition: no energy balance: no *Sperm analysis criteria:* WHO 5th edition *Number of semen samples:* Unspecified	Sperm volume Sperm concentration Total sperm number Sperm morphology (normal forms) DNA fragmentation Sperm motility (total motility, progressive, non-progressive) Round cells (number, positive correlation, negative correlation)

TT: total testosterone; FT: free testosterone; FSH: follicle stimulating hormone; LH: luteinizing hormone; C: cholesterol

^a^Sperm motility can be classified in different types. Type “a” are spermatozoa moving at > 20 mm–s, type “b” at 5–20 mm–s, type “c” at < 5 mm–s and type “d” are static

### Results of Individual Studies

#### Cross-Sectional Studies on Running

Three cross-sectional studies, including a total of 88 subjects, investigated the effect of endurance running on semen quality [30, 32, 34]. First, one study reported oligospermia in two out of 20 marathon runners. The mean sperm concentration in the other 18 men was 128 × 10^6^/ml (WHO 6th edition reference threshold for fertile men > 16 × 10^6^/ml) [30]. A second study compared 10 endurance runners with 8 weight lifters and 10 sedentary controls. Sperm concentration was lower in the runners group compared to sedentary controls (78 ± 12 × 10^6^/mL versus 176 ± 25 × 10^6^/ml, p = 0.003), but there were no differences in total sperm count. The runners presented with lower sperm progressive motility (40.8 ± 4.7% versus 58.7 ± 2.4% for controls, p < 0.05) and a lower number of morphologically normal sperm cells (40.2 ± 2.1% versus 47.0 ± 3.3% for controls, p < 0.05) [32]. A third study reported a reduction in sperm concentration (88.5 ± 14.8 × 10^6^/mL versus 175.5 ± 24.9 × 10^6^/ml, p = 0.045), total motile sperm count (134.5 ± 23.9 × 10^6^ versus 224.7 ± 39.1 × 10^6^, p = 0.037) and sperm motility (40.3 ± 4.3% progressive motility versus 58.7 ± 2.4%, p < 0.05) in high mileage runners compared to sedentary controls [34].

#### Longitudinal Studies on Running

Five longitudinal studies reporting on running were included, comprising a total of 109 subjects [19, 31, 33, 35, 36]. There were no significant alterations in semen parameters when investigating 12 endurance runners during 12 weeks [31]. A second study studied the effect of a two-week overtraining period in 5 endurance sportsmen (including running, swimming or cycling). Sperm concentration dropped immediately after overtraining and remained lower than baseline until three months afterwards (concentration before overtraining: 91 ± 23.3 × 10^6^/ml, immediately after: 52 ± 6.8 × 10^6^/ml, 3 months after overtraining 44.5 ± 20 × 10^6^/ml, p < 0.01). Sperm motility and morphology were not altered, though they included merely five athletes and the precise endurance sport they practiced was not enclosed [33]. A third study investigated the semen profile of 24 men, during a year in which they aimed to participate in a 56 km running competition. The marathon took place five months after the study started and further follow-up was performed until six months after the race. Training programs were progressively more intense in the pre-marathon period and gradually tapered off afterwards. Semen volume (p = 0.044) and sperm motility (p = 0.012) were lower four months after training started compared to baseline measurements. Sperm morphology was altered from one month after start of training until six months after the marathon (p < 0.05). There were no differences in sperm count (p > 0.05). When comparing high to low load training months, in high training months, a higher sperm count (133 × 10^6^/mL for high training and 71 × 10^6^/mL for low training, p = 0.001) and higher percentage of morphologically normal spermatozoa (15% for high training and 11% for low training, p = 0.001) was observed [35]. A fourth study followed 12 professional cyclists, nine elite triathletes and 10 marathon runners during a whole sports season. Semen analysis was performed three times: in the training, competition, and resting period. The investigators performed a mixed design study as they compared semen parameters between the groups, but also within groups during other periods. The results showed lower sperm motility in the runners compared to the cyclists in the resting period (p < 0.05) [19]. A fifth study group investigated the effect of increased training volume followed by a resting period on semen characteristics in eight runners. They did not observe significant group effects. However, two out of eight runners reached oligospermic values during periods of increased training, which spontaneously recovered to normal values after two weeks of rest [36].

To conclude, outcomes on sperm quality in endurance runners are mixed. Four studies did not report significant group effects [19, 30, 31, 36], while four other studies did find sperm alterations [32–35]. However, only one study described a decrease below the WHO thresholds, which may negatively affect fertility potential[35]. The decreases in semen quality reported by other authors were of statistical significance only as all semen parameters remained above WHO thresholds [32–34].

#### Cross-Sectional Studies on Cycling

Only one cross-sectional study reported on semen quality in endurance cyclists. Semen profiles of 10 non-professional cyclists showed no differences in sperm volume, motility, viability or count compared with 10 sedentary controls. Only morphological abnormalities were more frequent (normal morphology in cyclists 19.5 (18.3–30.8) % versus 41.5 (34.8–55.3) % in sedentary controls, p < 0.01) [10].

#### Longitudinal Studies on Cycling

Two longitudinal studies, including 46 subjects, assessed the effect of endurance cycling on semen parameters [19, 37]. First, during competition, Lucìa et al. reported reduced sperm motility in 12 cyclists compared to the other groups consisting of triathletes, marathon runners and sedentary controls (p < 0.05), but also when comparing with baseline values and during training periods (p < 0.01). After a resting period, the sperm motility of cyclists normalised and reached even higher values than the runners (p < 0.05). There were no anomalies in sperm morphology [19]. A second study group instructed biathletes to double cycling hours for two weeks without changing cycling intensity or running volume. Oligospermia was noticed in one subject. However, this study was limited by the small sample size, as only six athletes were included and single semen analysis was performed [37].

#### Cross-Sectional Studies on Triathlon

One study group conducted three cross-sectional studies on semen quality in triathletes[38–40]. In the first article, they compared the semen profile of 15 professional triathletes with 14 waterpolo players and 16 physically active men who practiced different ball sports[38]. Total sperm count (141.3 ± 58.0 × 106 in triathletes, versus 191.8 ± 73.4 × 10^6^ ball sports and 196.6 ± 85.4 × 10^6^ for water polo players, p = 0.03) and concentration (48.2 ± 14.7 × 10^6^/mL in triathletes versus 61.0 ± 23.0 × 10^6^/mL in ball sports and 58.0 ± 24.4 × 10^6^/mL in water polo players, p = 0.04) of the triathletes were lower compared to both other groups. Three triathletes even reached oligospermic levels. Sperm morphology was significantly altered compared to both other groups (4.7 ± 2.2% normal forms in triathletes versus 15.2 ± 1.2% in ball sports and 9.7 ± 3.0% in water polo players, p = 0.01). Moreover, in some subjects the number of morphologically normal forms decreased to < 4%, a critical level for Kruger’s strict criteria[41]. For further investigation of this outcome, the relationship between training volume and sperm morphology was investigated in a second study [40]. Although not specified by the authors, we assume data from the same triathletes as in the first study were examined. The results showed no correlation between normal forms and total weekly volume, or between running or swimming mileage (p > 0.05). In contrast, cycling kilometres negatively correlated with normal sperm morphology (p < 0.05). More recently, the same authors carried out a third study in 12 high-level triathletes [39]. Semen analysis showed no abnormal values. However, mean morphology was in the lower margins of normality, with some subjects reaching the < 4% threshold of Kruger’s strict criteria. Besides conventional semen parameters, DNA fragmentation was examined as well and showed higher values, even above the WHO threshold (p < 0.05).

#### Longitudinal Studies on Triathlon

Only one study reported longitudinal data on seminal characteristics of triathletes and used a mixed design. In contrast with the cross-sectional studies mentioned above, semen parameters did not change over time and remained normal during the whole sports season [19].

## Discussion

### Summary of Evidence

In this systematic review, we found that data on the effect of endurance exercise on semen quality is inconsistent. Most studies found no [19, 30, 31, 36, 37] or only subclinical group effects [10, 19, 32–34]. When statistically significant differences in semen parameters were observed, absolute values remained above WHO-defined thresholds. However, the limited number of participants and sedentary controls hamper interpretation of the results. Endurance sports alone do not seem to critically disrupt spermatogenesis. However, they could be a decisive factor in men who already have low or low-normal sperm quality. Four trials reported a decrease in certain parameters of semen quality with potential clinical consequences [35, 38–40]. The amount of morphologically normal sperm cells was reduced below the threshold of Kruger’s strict criteria in some cyclists and triathletes[35, 38, 40]. Also, sperm DNA fragmentation was higher in triathletes [39].

Endurance exercise may also have long-term effects on semen quality. Comparing with baseline semen analysis, two longitudinal studies reported a statistically significant reduction in sperm concentration and morphology three months after overtraining and eight months after running a marathon, respectively [33, 35]. These long-term alterations may be a reflection of the 70 days to complete spermatogenesis, as germ cells could be damaged at the start of this process. It is unclear to what extent these findings affect clinical fertility potential, since recent literature did not find any association between isolated low percentage of sperm morphology and pregnancy rate [42]. High sperm DNA fragmentation can have a potential negative influence on fertility. However, since there are no recommendations on cut-off values or standard measuring techniques, the clinical relevance remains unclear [16, 43]. In general, sperm concentration and motility are the most important parameters with respect to pregnancy rate [42]. Since none of the included studies observed changes in these parameters, our study suggests that endurance exercise has no major impact on fertility potential. Interestingly, a recent retrospective study observed that male professional soccer players fathered more girls than boys. Of the 122 children born, there were 52 boys (42.6%) versus 70 girls (57.4%) and differences in training volume and intensity significantly impacted the birth offspring ratio more towards females [44]. Endurance exercise could thus potentially impact reproductive parameters in a more complex way, warranting more research on underlying mechanisms and effects.

Several mechanisms have been suggested to explain how endurance exercise could affect semen quality. A recurring hypothesis is the “volume threshold”, which suggested that a certain training load for running (> 104 km/w) or cycling (> 300 km/w) negatively affects sperm quality [34, 40]. This hypothesis can be questioned, because the cycling threshold was based on morphological alterations and other studies could not find significant sperm alterations despite attaining these thresholds [19, 36]. In contrast to training load, exercise intensity can be more important [35]. However, neither training load nor exercise intensity can fully explain the underlying mechanism, as multiple factors may influence spermatogenesis. Both reproductive hormone levels and BMI remained in the normal range in all of the included studies that reported these parameters [45]. In addition to BMI, energy balance should be examined more thoroughly to obtain more insight on LEA. In one study professional cyclists exhibited altered sperm motility [19]. However, since mean testosterone levels and body fat percentage remained normal, it is unlikely that hormonal suppression or undernutrition are responsible for these sperm alterations.

To date, this field of research faces many methodological challenges. First of all, the term “endurance exercise” is open to interpretation because there is no consensus on minimal training hours or volume to be attained. Second, setting up a standardised study design is complicated because various factors such as scrotal temperature, saddle position, training load, intensity and energy balance should be controlled. Third, there is a lack of standardisation in semen analysis. For example, one study in endurance runners had a time delay of more than 24 h between sample production and analysis, whereas sample production and analysis should be performed within one hour [31]. Among the included studies, there are also important differences between the number of semen samples analysed and abstinence time is not always disclosed. This is of significance because higher abstinence time is associated with lower motility and higher DNA damage. To account for individual variability in semen parameters, two consecutive semen samples should be examined [24, 26]. Furthermore, because of the small number of participants and the absence of a control group in several studies, it is unclear if observed differences in semen characteristics are merely due to normal variation or indeed induced by exercise.

In recent decades, there have been multiple guidelines made available regarding examination of human spermatozoa (Supplementary Table S6) [46]. This resulted in heterogeneous results and interpretations, but also in difficulties in comparing studies. Moreover**,** recent literature showed a lack of adherence to the standardised WHO guidelines for semen analysis [47]. Inadequate methodology causes measurement uncertainty with inaccurate results. Therefore, the reproducibility and reliability of previously published data can be questioned [25]. Finally, some of the included studies were rather old and a notable proportion were carried out by the same authors, especially regarding triathlon. To address these methodological concerns, future researchers are recommended to follow the laboratory methods and corresponding checklist proposed by Björndahl et al. in 2022 [25].

### Strengths and Limitations

This systematic review has certain strengths. Because of our detailed search strategy, we conducted a comprehensive search for evidence, which enabled us to systematically evaluate the impact of endurance exercise on semen quality of male athletes. To avoid reporting bias, two investigators independently screened the obtained articles for eligibility. A formal assessment of bias in the included evidence was conducted using an approved tool. To report transparently, this systematic review was written following the PRISMA 2020 statement (Supplementary Appendix S1) [27].

Our study has also some limitations. Data extraction was done by one reviewer. Even though this research domain covers a long time period, the number of eligible studies was small. Some of the studies were rather old. The majority lacked methodological quality and had a small sample size. To assess semen quality, each parameter was evaluated separately. However, it is possible that a combination of multiple disrupted parameters (e.g., low concentration together with low progressive motility) may have an effect on reproductive function. Moreover, when assessing sperm parameters, interobserver variation for morphology, for example, is larger than for concentration. This adds to the difficulty comparing between studies.

## Conclusion

This systematic review shows that endurance exercise can have a negative effect on semen quality, although rarely with clinical relevance on fertility potential. In general, semen parameters, especially concentration and motility, remained above WHO defined thresholds. The obtained data were highly affected by small sample sizes and methodological pitfalls, which may have led to measurement uncertainty. Therefore, there is a need for future research of high methodological quality to further assess the relationship between endurance exercise and semen quality.

### Supplementary Information

Additional file1 (PDF 1355 kb)

## Data Availability

Data sharing is not applicable to this article as no datasets were generated or analyzed during the current study.

## Abbreviations

WHO: World Health Organisation
LEA: Low energy availability
HPG: Hypogonadal-pituitary–gonadal axis
GnRH: Gonadotropin-releasing hormone
TSH: Thyroid-stimulating hormone
LH: Luteinizing hormone
REDs: Relative energy deficiency in sport
ROS: Reactive oxygen species
AAS: Anabolic androgenic steroids
PRISMA: Preferred Reporting Items for Systematic reviews and Meta-Analyses
MET: Metabolic equivalent of task
