# Supplementary material for: The Effect of Endurance Exercise on Semen Quality in Male Athletes: A Systematic Review
Source: Sports Med Open. 2024 Jun 11;10:72. doi: 10.1186/s40798-024-00739-z (PMC11166609; doi:10.1186/s40798-024-00739-z)
Supplement: Supplementary file 1 — Additional file1 (PDF 1355 kb) [file 40798_2024_739_MOESM1_ESM.pdf]

The effect of endurance exercise on semen quality in male athletes: a systematic review

Alex Aerts[1*], Annelien Temmerman[1,2*], Arne Vanhie[3], Dirk Vanderschueren[4,5], Leen Antonio[4,5 **]

*Shared first author

Affiliations:

1. Faculty of Medicine, Katholieke Universiteit Leuven, Leuven, Belgium
2. Department of Internal Medicine, University Hospitals Leuven, Leuven, Belgium
3. Leuven University Fertility Centre, University Hospitals Leuven, Leuven, Belgium
4. Clinical and Experimental Endocrinology, Department of Chronic Diseases and Metabolism, KU Leuven, Leuven, Belgium
5. Department of Endocrinology, University Hospitals Leuven, Belgium

**** Corresponding author:**

Leen Antonio, MD PhD

Laboratory of Clinical and Experimental Endocrinology

Department of Chronic Diseases and Metabolism (CHROMETA)

University of Leuven - Campus GHB O/N 1

Herestraat 49 box 902

B-3000 Leuven

Belgium

Email: leen.antonio@kuleuven.be

ORCID: 0000-0002-1079-2860

Table S1: Complete search strategy per database.

MEDLINE (PubMed)

Concept 1: semen quality

"Semen Analysis"[Mesh] OR "Testis"[Mesh] OR "testis"[tiab] OR "testes"[tiab] OR "testic*"[tiab] OR "Leydig Cell*"[tiab] OR "Testicular interstitial cell*"[tiab] OR "rete testis"[tiab] OR "seminiferous tubule*"[tiab] OR "Sertoli cell*"[tiab] OR "Semen"[Mesh] OR "semen"[tiab] OR "seminal"[tiab] OR "Infertility, Male"[Mesh] OR "infertility"[tiab] OR "aspermia"[tiab] OR "asthenozoospermia"[tiab] OR "asthenoteratozoospermia*"[tiab] OR "azoospermia"[tiab] OR "oligospermia"[tiab] OR "cryptozoospermia"[tiab] OR "male sterility"[tiab] OR "spermatogenic failure"[tiab] OR "hypospermatogenes*"[tiab] OR "oligoasthenoteratozoospermia*"[tiab] OR "oligozoospermia"[tiab] "teratozoospermia*"[tiab] OR "teratospermia*"[tiab] OR "globozoospermia*"[tiab] OR "sperm"[tiab] OR "fertility"[tiab] OR "reproductive function"[tiab] OR "reproduction"[tiab] OR "HPT axis"[tiab] OR "hypogonadism"[tiab] OR "Hypogonadism"[Mesh:NoExp] OR "hypogonadotropic hypogonadism"[tiab]

AND

Concept 2: exercise

"Exercise"[Mesh:NoExp] OR "exercise*"[tiab] OR "Endurance Training"[Mesh] OR "endurance training"[tiab] OR "Physical Endurance"[Mesh:NoExp] OR "physical endurance"[tiab] OR "Athletes"[Mesh] OR "athlet*"[tiab] OR "olympi*"[tiab] OR "Physical Exertion"[Mesh] OR "physical exertion"[tiab] OR "physical effort*"[tiab] OR "physical activit*"[tiab] OR "workout"[tiab] OR "work-out"[tiab] OR "Running"[Mesh:NoExp] OR "runner*"[tiab] OR "running*"[tiab] OR "Marathon Running"[Mesh] OR "marathon*"[tiab] OR "ultramarathon*"[tiab] OR "Swimming"[Mesh] OR "swimming"[tiab] OR "Bicycling"[Mesh] OR "bicycling"[tiab] OR "cycling"[tiab] OR "cyclist*"[tiab] OR "endurance"[tiab] OR "sport*"[tiab] OR "Sports"[Mesh:NoExp]

Embase

Concept 1: semen quality

'semen analysis'/exp OR 'testis'/exp OR 'testis':ti,ab,kw OR 'testes':ti,ab,kw OR 'testic*':ti,ab,kw OR 'Leydig Cell*':ti,ab,kw OR 'Testicular interstitial cell*':ti,ab,kw OR 'rete testis':ti,ab,kw OR 'seminiferous tubule*':ti,ab,kw OR 'Sertoli cell*':ti,ab,kw OR 'sperm'/exp OR 'sperm':ti,ab,kw OR 'semen':ti,ab,kw OR 'seminal':ti,ab,kw OR 'male infertility'/exp OR 'infertility':ti,ab,kw OR 'aspermia':ti,ab,kw OR 'asthenozoospermia':ti,ab,kw OR 'asthenoteratozoospermia*':ti,ab,kw OR 'azoospermia':ti,ab,kw OR 'oligospermia':ti,ab,kw OR 'cryptozoospermia':ti,ab,kw OR 'male sterility':ti,ab,kw OR 'spermatogenic failure':ti,ab,kw OR 'hypospermatogenes*':ti,ab,kw OR 'oligoasthenoteratozoospermia*':ti,ab,kw OR 'oligozoospermia':ti,ab,kw 'teratozoospermia*':ti,ab,kw OR 'teratospermia*':ti,ab,kw OR 'spermatozoon abnormality':ti,ab,kw OR 'globozoospermia*':ti,ab,kw OR 'fertility':ti,ab,kw OR 'reproduction':ti,ab,kw OR 'reproductive function':ti,ab,kw OR 'HPT axis':ti,ab,kw OR 'hypogonadism':ti,ab,kw OR 'hypogonadism'/exp OR 'hypogonadotropic hypogonadism'/exp OR 'hypogonadotropic hypogonadism':ti,ab,kw

AND

Concept 2: exercise

'exercise'/de OR 'exercise*':ti,ab,kw OR 'endurance training'/exp OR 'endurance training':ti,ab,kw OR 'endurance'/exp OR 'endurance':ti,ab,kw OR 'athlete'/exp OR 'athlet*':ti,ab,kw OR 'elite athlete'/exp OR 'olympi*':ti,ab,kw OR 'physical exertion':ti,ab,kw OR 'physical effort*':ti,ab,kw OR 'physical activit*':ti,ab,kw OR 'workout':ti,ab,kw OR 'work-out':ti,ab,kw OR 'running'/exp OR 'running*':ti,ab,kw OR 'runner'/exp OR 'runner*':ti,ab,kw OR 'marathon*':ti,ab,kw OR 'ultramarathon*':ti,ab,kw OR 'swimming'/exp OR 'swimming':ti,ab,kw OR 'cycling'/exp OR 'bicycling':ti,ab,kw OR 'cycling':ti,ab,kw OR 'cyclist'/exp OR 'cyclist*':ti,ab,kw OR 'sport*':ti,ab,kw OR 'sport'/de

SPORTDiscus

Concept 1: semen quality

"Semen Analysis" OR "testis" OR "testes" OR "testic*" OR "Leydig Cell*" OR "Testicular interstitial cell*" OR "rete testis" OR "seminiferous tubule*" OR "Sertoli cell*" OR "Semen" OR "seminal" OR "Infertility, Male" OR "infertility" OR "aspermia" OR "asthenozoospermia" OR "asthenoteratozoospermia*" OR "azoospermia" OR "oligospermia" OR "cryptozoospermia" OR "male sterility" OR "spermatogenic failure" OR "hypospermatogenes*" OR "oligoasthenoteratozoospermia*" OR "oligozoospermia" "teratozoospermia*" OR "teratospermia*" OR "globozoospermia*" OR "sperm" OR "fertility" OR "reproductive function" OR "reproduction" OR "HPT axis" OR "hypogonadism" OR "hypogonadotropic hypogonadism"

AND

Concept 2: exercise

"exercise*" OR "endurance training" OR "physical endurance" OR "athlet*" OR "olympi*" OR "physical exertion" OR "physical effort*" OR "physical activit*" OR "workout" OR "work-out" OR "runner*" OR "running*" OR "marathon*" OR "ultramarathon*" OR "swimming" OR "bicycling" OR "cycling" OR "cyclist*" OR "endurance" OR "sport*"

Cochrane Central Register of Controlled Trials (CENTRAL)

Concept 1: semen quality

[mh "Semen Analysis"] OR [mh Testis] OR ("testis" OR "testes" OR testic* OR (Leydig NEXT Cell*) OR (Testicular NEXT interstitial NEXT cell*) OR "rete testis" OR (seminiferous NEXT tubule*) OR (Sertoli NEXT cell*)):ti,ab,kw OR [mh Semen] OR ("semen" OR "seminal"):ti,ab,kw OR [mh "Infertility, Male"] OR ("infertility" OR "aspermia" OR "asthenozoospermia" OR asthenoteratozoospermia* OR "azoospermia" OR "oligospermia" OR "cryptozoospermia" OR "male sterility" OR "spermatogenic failure" OR hypospermatogenes* OR oligoasthenoteratozoospermia* OR "oligozoospermia" OR teratozoospermia* OR teratospermia* OR globozoospermia* OR "sperm" OR "fertility" OR "reproductive function" OR

“reproduction” OR “HPT axis” OR “hypogonadism” OR (“hypogonadotropic NEXT hypogonadism”)):ti,ab,kw OR [mh ^Hypogonadism]
AND
<u>Concept 2: exercise</u>
[mh ^Exercise] OR (exercise*):ti,ab,kw OR [mh "Endurance Training"] OR (endurance training):ti,ab,kw OR [mh ^”Physical Endurance”] OR (physical endurance):ti,ab,kw OR [mh Athletes] OR (athlet* OR olympi*):ti,ab,kw OR [mh "Physical Exertion"] OR ((“physical exertion”) OR (physical NEXT effort*) OR (physical NEXT activit*) OR “workout” OR “work-out”):ti,ab,kw OR [mh ^Running] OR (runner* OR running*):ti,ab,kw OR [mh "Marathon Running"] OR (marathon* OR ultramarathon*):ti,ab,kw OR [mh "Swimming"] OR (“swimming”):ti,ab,kw OR [mh "Bicycling"] OR (“bicycling” OR “cycling” OR cyclist* OR “endurance” OR sport*):ti,ab,kw OR [mh ^Sports]

Clinicaltrials.gov

<u>Condition or disease</u>
"Semen Analysis" OR “testis” OR “testes” OR “testic” OR “Leydig Cell” OR “Testicular interstitial cell” OR “rete testis” OR “seminiferous tubule” OR “Sertoli cell” OR "Semen" OR “seminal” OR "Infertility, Male" OR “infertility” OR “aspermia” OR “asthenozoospermia” OR “asthenoteratozoospermia” OR “azoospermia” OR “oligospermia” OR “cryptozoospermia” OR “male sterility” OR “spermatogenic failure” OR “hypospermatogenes” OR “oligoasthenoteratozoospermia” OR “oligozoospermia” “teratozoospermia” OR “teratospermia” OR “globozoospermia” OR “sperm” OR “fertility” OR “reproductive function” OR “reproduction” OR “HPT axis” OR “hypogonadism” OR “hypogonadotropic hypogonadism”
<u>Other terms</u>
“exercise” OR “endurance training” OR “physical endurance” OR “athlete” OR “athletes” OR “olympic” OR “Olympian” OR “physical exertion” OR “physical effort” OR “physical activity” OR “workout” OR “work-out” OR “runner” OR “runners” OR “running” OR “marathon” OR “ultramarathon” OR “swimming” OR “bicycling” OR “cycling” OR “cyclist” OR “cyclists” OR “endurance” OR “sport”

International Clinical Trials Registry Platform (ICTRP)

<u>Concept 1: semen quality</u>
Semen Analysis OR testis OR testes OR testic OR Leydig Cell OR Testicular interstitial cell OR rete testis OR seminiferous tubule OR Sertoli cell OR Semen OR seminal OR Infertility, Male OR infertility OR aspermia OR asthenozoospermia OR asthenoteratozoospermia OR azoospermia OR oligospermia OR cryptozoospermia OR male sterility OR spermatogenic failure OR hypospermatogenes OR oligoasthenoteratozoospermia OR oligozoospermia teratozoospermia OR teratospermia OR globozoospermia OR sperm OR fertility OR reproductive function OR reproduction OR HPT axis OR hypogonadism OR hypogonadotropic hypogonadism
AND
<u>Concept 2: exercise</u>
exercise OR endurance training OR physical endurance OR athlete OR athletes OR olympic OR Olympian OR physical exertion OR physical effort OR physical activity OR workout OR work-out OR runner OR runners OR running OR marathon OR ultramarathon OR swimming OR bicycling OR cycling OR cyclist OR cyclists OR endurance OR sport

Table S2. Characteristics of included studies investigating endurance running.

Author, year, country	Study design	Participants	Age (years)	Intervention	Methods	Outcomes			Summary of results
Ayers et al. (1985) USA	Cross-sectional	Endurance runners (n=20)<br>(48-129 km/w)<br><br>Sedentary controls (n=10)	26-42	/	*Other assessments:*<br>- hormonal: yes<br>TT, FT, LH, DHEA-S, E_2, PRL<br>- body composition: yes<br>- energy balance: no<br><br>*Sperm analysis criteria:*<br>Unspecified<br><br>*Number of semen samples:*<br>1	Sperm count ($p < 0.05$):<br>- oligospermia in 2 runners: < 15 x 10^6/ml<br>- mean count in other subjects: 128 x 10^6/ml<br><br>Sperm morphology ($p > 0.05$):<br>- no difference			Endurance running does not negatively affect sperm quality.
Bagatell et al. (1990) USA	Longitudinal	Endurance runners (R) (n=12)<br>(> 64 km/w)<br><br>Sedentary controls (SC) (n=12)	21-37	/	*Other assessments:*<br>- hormonal: yes<br>TT, FT, SHBG, FSH, LH, C<br>- body composition: yes<br>- energy balance: no<br><br>*Sperm analysis criteria:*<br>Bremner et al. (1981)<br><br>*Number of semen samples:*<br>6 (1 at 2-week intervals for 12 weeks)	Sperm count ($p > 0.05$):<br>- R: 119.9 ± 64.4 x 10^6/ml<br>- SC: 108.9 ± 91.7 x 10^6/ml<br><br>Total spermatozoa per ejaculate ($p > 0.05$):<br>- R: 436.8 ± 64.6 x 10^6<br>- SC: 316.1 ± 79.8 x 10^6	Sperm motility<br>*Forward progressive* ($p > 0.05$):<br>- R: 82.0 ± 4.6 %<br>- SC: 73.2 ± 3.5 %<br><br>Sperm morphology<br>*Oval forms* ($p > 0.05$):<br>- R: 81.1 ± 1.8 %<br>- SC: 78.9 ± 2.7 %		Endurance running does not negatively affect sperm quality.
Arce et al. (1993) USA	Cross-sectional	Endurance runners (R) (n=10)<br>(109.2 ± 4.8 km/w)<br><br>Sedentary controls (SC) (n=10)<br>(< 1h/w)<br><br>Resistance-trained weightlifters (WL) (n=8)<br>(> 2 h/x, > 4 x/w)	18-35	/	*Other assessments:*<br>- hormonal: yes<br>TT, FT, LH, FSH, E_2, PRL<br>- body composition: yes<br>- energy balance: no<br><br>*Sperm analysis criteria:*<br>WHO 2nd edition<br><br>*Number of semen samples:*<br>2-5	Sperm volume ($p = 0.086$):<br>- R: 4.2 ± 0.5 ml<br>- WL: 3.0 ± 0.5 ml<br>- SC: 2.5 ± 0.5 ml<br><br>Sperm density ($p = 0.003$):<br>- R: 78 ± 12 x 106/ml (lower vs SC)<br>- WL: 122 ± 15 x 106/ml<br>- SC: 176 ± 25 x 106/ml<br><br>Total sperm count ($p = 0.858$):<br>- R: 332 ± 74 x 10^6<br>- WL: 342 ± 36 x 10^6<br>- SC: 376 ± 59 x 10^6<br><br>In vitro penetration of bovine CM ($p = 0.036$):<br>- R: 22 ± 5 mm Penetrak (lower vs SC)<br>- WL: /<br>- SC: [illegible] ± 7 mm Penetrak	Sperm motility<br>*Forward progressive* ($p = 0.005$):<br>- R: 40.8 ± 4.7 % (lower vs WL and SC)<br>- WL: 58.0 ± 4.6 %<br>- SC: 58.7 ± 2.4 %<br>*Non-progressive* ($p = 0.174$):<br>- R: 5.0 ± 1.0 %<br>- WL: 2.8 ± 1.5 %<br>- SC: 2.0 ± 1.0 %<br>*Non-motile* ($p = 0.011$):<br>- R: 54.2 ± 4.9 % (higher vs WL and SC)<br>- WL: 39.2 ± 3.9 %<br>- SC: 39.3 ± 1.9 %	Sperm morphology<br>*Normal* ($p = 0.006$):<br>- R: 40.2 ± 2.1 % (lower vs WL)<br>- WL: 54.8 ± 2.9 %<br>- SC: 47.0 ± 3.3 %<br>*Immature* ($p = 0.035$):<br>- R: 17.2 ± 2.4 % (higher vs WL and SC)<br>- WL: 10.5 ± 2.1 %<br>- SC: 10.9 ± 1.2 %<br>*Round cells* ($p = 0.001$):<br>- R: 8.3 ± 1.7 % (higher vs WL and SC)<br>- WL: 0.6 ± 0.4 %<br>- SC: 2.5 ± 0.9 %	Endurance running is associated with subclinical modifications in multiple sperm parameters.
Roberts et al. (1993) USA	Longitudinal	Endurance-trained men (n=5)<br>(running, swimming and cycling)<br>(> 4 d/w)	23-26	Doubling weekly mileage at constant intensity<br>(2 weeks)	*Other assessments:*<br>- hormonal: yes<br>TT, C<br>- body composition: yes<br>- energy balance: yes<br><br>*Sperm analysis criteria:*<br>Unspecified<br><br>*Number of semen samples:*<br>- 4 before overtraining (6-8 week intervals)<br>- 1 immediately after overtraining<br>- 1 after 3 months	Sperm concentration ($p < 0.01$):<br>- before overtraining: 91 ± 23.3 x 10^6/ml<br>- immediately after: 52 ± 6.8 x 10^6/ml<br>- 3 months after overtraining 44.5 ± 20 x 10^6/ml<br><br>Sperm morphology and sperm motility:<br>- no difference (no p-values or data disclosed)			Endurance exercise induces a subclinical decrease in fertility potential as sperm concentration was decreased immediately after overtraining, but also 3 months later.

De Souza et al. (1994)<br>USA	Cross-sectional	High mileage endurance runners (HR) (n=11)<br>(108.0 ± 4.5 km/w)<br><br>Moderate mileage runners (MR) (n=9)<br>(54.2 ± 3.7 km/w)<br><br>Sedentary controls (SC) (n=10)<br>(< 1 h/w)	18-35	/	*Other assessments:*<br>- hormonal: yes<br>TT, FT, LH, FSH, PRL, ACTH, C, DHEA-SO_4, T_3, FT_3, T_4, FT_4<br>- body composition: yes<br>- energy balance: yes<br><br>*Sperm analysis criteria:*<br>WHO 2nd edition<br><br>*Number of semen samples:*<br>2-5	Sperm volume (p = 0.116):<br>- HR: 4.1 ± 0.5 ml<br>- MR: 3.5 ± 0.6 ml<br>- SC: 2.5 ± 0.5 ml<br><br>Sperm density (p = 0.045):<br>- HR: 88.5 ± 14.8 x 10^6/ml (lower vs SC)<br>- MR: 127.2 ± 32.2 x 10^6/ml<br>- SC: 175.5 ± 24.9 x 10^6/ml<br><br>Sperm count (p = 0.858):<br>- HR: 352.0 ± 69.6 x 10^6<br>- MR: 317.8 ± 83.3 x 10^6<br>- SC: 375.9 ± 58.9 x 10^6<br><br>Normal motile count (p = 0.052):<br>- HR: 58.5 ± 10.8 x 10^6 (lower vs MR and SC)<br>- MR: 118.8 ± 20.3 x 10^6<br>- SC: 106.7 ± 22.3 x 10^6<br><br>Motile count (p = 0.037):<br>- HR: 134.5 ± 23.9 x 10^6 (lower vs SC)<br>- MR: 240.1 ± 45.3 x 10^6<br>- SC: 224.7 ± 39.1 x 10^6	Sperm penetration of cervical mucus (p = 0.024):<br>- HR: 26.8 ± 6.3 mm Penetrak<br>- MR: 37.5 ± 7.2 mm Penetrak<br>- SC: 43.2 ± 7.0 mm Penetrak<br><br>Sperm motility<br>*Forward progressive* (p = 0.006):<br>- HR: 40.3 ± 4.3 % (lower vs SC)<br>- MR: 48.8 ± 4.5 % (lower vs SC)<br>- SC: 58.7 ± 2.4 %<br>*Non-progressive* (p = 0.014):<br>- HR: 6.1 ± 1.4 % (higher vs SC)<br>- MR: 7.3 ± 1.1 % (higher vs SC)<br>- SC: 2.0 ± 1.0 %<br>*Non-motile* (p = 0.023):<br>- HR: 53.6 ± 4.4 % (higher vs SC)<br>- MR: 43.9 ± 3.8 %<br>- SC: 39.3 ± 1.9 %	Sperm morphology<br>*Normal* (p = 0.341):<br>- HR: 40.9 ± 2.0 %<br>- MR: 46.4 ± 4.4 %<br>- SC: 47.0 ± 3.3 %<br>*Large* (p = 0.760):<br>- HR: 2.5 ± 0.8 %<br>- MR: 1.7 ± 0.6 %<br>- SC: 2.3 ± 1.0 %<br>*Small* (p = 0.350):<br>- HR: 3.9 ± 0.7 %<br>- MR: 2.5 ± 1.1 %<br>- SC: 2.3 ± 0.5 %<br>*Amorphous* (p = 0.653):<br>- HR: 34.5 ± 2.3 %<br>- MR: 37.2 ± 2.2 %<br>- SC: 37.4 ± 2.8 %<br>*Immature* (p = 0.031):<br>- HR: 16.8 ± 2.2 % (higher vs MR and SC)<br>- MR: 10.1 ± 2.0 %<br>- SC: 10.9 ± 1.2 %<br>*Round cells* (p = 0.004):<br>- HR: 8.0 ± 1.6 % (higher vs MR and SC)<br>- MR: 2.5 ± 1.1 %<br>- SC: 2.5 ± 0.9 %	High volume endurance running (> 104 km/w) is associated with sublclinical alterations in semen profile.
Jensen et al. (1995)<br>South Africa	Longitudinal	Endurance runners (n=24)	25-54	High training months:<br>60-160 km/w<br><br>Low training months:<br>< 55 km/w<br><br>(over a 12 months period)	*Other assessments:*<br>- hormonal: yes<br>TT, LH, FSH, PRL, E_2 , P<br>- body composition: yes<br>- energy balance: no<br><br>*Sperm analysis criteria:*<br>Kruger's strict criteria (1986) for morphology<br><br>*Number of semen samples:*<br>1 in each of the following months: December, January, February, April, May, August, November	Sperm volume<br>*Training load* (p = 0.532):<br>- high: 2.7 ± 1.4 ml<br>- low: 2.8 ± 1.4 ml<br>*Period* (p = 0.044):<br>decrease in April<br><br>Sperm count<br>*Training load* (p = 0.001):<br>- high: 133 ± 141 x 10^6/ml<br>- low: 71 ± 65 x 10^6/ml<br>*Period* (p > 0.05):<br>no change during whole study	Sperm morphology (normal forms)<br>*Training load* (p = 0.001):<br>- high: 15 ± 6 %<br>- low: 11 ± 7 %<br>*Period:*<br>decrease in January (p = 0.011), February (p = 0.008), April (p = 0.016), May (p = 0.005), August (p = 0.021) and November (p = 0.001)<br><br>Sperm motility<br>*Training load* (p = 0.610):<br>- high: 54 ± 9 %<br>- low: 55 ± 8 %<br>*Period* (p = 0.012):<br>decrease in April		Endurance running negatively affects fertility because sperm morphology is clinically altered in preparation for a marathon, but also 8 months afterwards.
Lucía et al. (1996)<br>Spain	Longitudinal & cross-sectional	Professional cyclists (C) (n=12)<br>(884.46 ± 44.7 km/w)<br><br>Elite triathletes (TA) (n=9)<br>- running: 54.36 ± 7.2 km/w<br>- swimming: 14.36 ± 5.8 km/w<br>- cycling: 316.16 ± 79.0 km/w<br><br>Marathon runners (R) (n=10)<br>(94.26 ± 27.1 km/w)<br><br>Sedentary controls (n=9)	22-38	Follow-up during one sports season:<br>- precompetition<br>- competition<br>- resting period (2 weeks)	*Other assessments:*<br>- hormonal: yes<br>TT, FT, FSH, LH, C<br>- body composition: yes<br>- energy balance: no<br><br>*Sperm analysis criteria:*<br>WHO 3rd edition<br><br>*Number of semen samples:*<br>- 1 in precompetition<br>- 1 in competition<br>- 1 after resting period	Semen morphology, volume and density (p > 0.05):<br>- no differences between or within groups<br><br>Sperm motility<br>*Competition* :<br>- lower in C vs R and TA (p < 0.05)<br>- lower in C vs themselves during the other two periods (p < 0.01)<br>*Resting period:*<br>- higher in C vs R (p < 0.05)<br><br>Note: absolute data were not mentioned, but depicted in graphics.			Endurance running does not negatively affect sperm quality.
Hall et al. (1999)<br>USA	Longitudinal	Endurance runners (n=8)<br>(32-64 km/w)<br><br>Sedentary controls (n=8)	19-37	2 weeks normal training (NT)<br><br>2 weeks at 143% NT (IT1)<br><br>2 weeks at 186% NT (IT2)<br><br>2 weeks at 50% NT (RT)	*Other assessments:*<br>- hormonal: yes<br>TT, FT, FSH, LH, PRL, C<br>- body composition: no<br>- energy balance: no<br><br>*Sperm analysis criteria:*<br>Bremner et al. (1981)<br><br>*Number of semen samples:*<br>- 1 after 2 weeks NT<br>- 1 after 2 weeks IT1<br>- 1 after 2 weeks IT2<br>- 1 after 2 weeks RT	Sperm motility and morphology (p > 0.05):<br>- no difference<br><br>Sperm count (p > 0.05):<br>- mean sperm count: no difference<br>- oligospermia in 2 runners<br>*Runner 1:*<br>- NT: 314 x 10^6<br>- IT1 and IT2: 17 x 10^6<br>- RT: 132 x 10^6<br>*Runner 7:*<br>- NT: 134 x 10^6<br>- IT1: 108 x 10^6<br>- IT2: 31 x 10^6<br>- RT: 165 x 10^6<br><br>Note: absolute data were not mentioned but depicted in graphics.			Four weeks of increased training volume and two weeks of reduced training volume does not negatively affect sperm quality in endurance runners.

TT: total testosterone; FT: free testosterone; LH: luteinizing hormone; DHEA-S: dehydroepiandrosterone-sulfate; E_2: estradiol; PRL: prolactin; SHBG: sex hormone binding globulin; FSH: follicle stimulating hormone; C: cholesterol; ACTH: adrenocorticotropic hormone; DHEA-SO_4: dehydroepiandrosterone-sulfate; T_3: total triiodothyronine, FT_3: free triiodothyronine; T_4: thyroxine, FT_4: free thyroxine; P: phosphate;

Table S3. Characteristics of literature found on cycling.

Author, year, country	Study design	Participants	Age (years)	Intervention	Methods	Outcomes	Summary of results
Griffith et al.<br>(1990)<br>USA	Longitudinal	Biathletes (n=6):<br>- running: 40-65 km/w<br>- cycling: 85-190 km/w<br>- weightlifting: 2-3x/w	22-44	Double bicycling hours (2 weeks)	*Other assessments:*<br>- hormonal: yes<br>TT<br>- body composition: yes<br>- energy balance: no<br><br>*Sperm analysis criteria:*<br>Unspecified<br><br>*Number of semen samples:*<br>- 1 baseline<br>- 1 in follow-up	Sperm count (p > 0.05):<br>- before overtraining: 108 ± 56 x 10^6/ml<br>- after overtraining: 77 ± 61 x 10^6/ml<br>- oligospermia in 1 subject: decrease from 49 to 6 x 10^6/ml	Increasing training volume has no significant effect on sperm quality.
Lucía et al.<br>(1996)<br>Spain	Longitudinal & cross-sectional	Professional cyclists (C) (n=12)<br>(884.46 ± 44.7 km/w)<br><br>Elite triathletes (TA) (n=9)<br>- running: 54.36 ± 7.2 km/w<br>- swimming: 14.36 ± 5.8 km/w<br>- cycling: 316.16 ± 79.0 km/w<br><br>Marathon runners (R) (n=10)<br>(94.26 ± 27.1 km/w)<br><br>Sedentary controls (SC) (n=9)	22-38	Follow-up during one sports season:<br>- precompetition<br>- competition<br>- resting period (2 weeks)	*Other assessments:*<br>- hormonal: yes<br>TT, FT, FSH, LH, C<br>- body composition: yes<br>- energy balance: no<br><br>*Sperm analysis criteria:*<br>WHO 3rd edition<br><br>*Number of semen samples:*<br>- 1 in pre-competition<br>- 1 in competition<br>- 1 after resting period	Semen morphology, volume and density (p > 0.05):<br>- no differences between or within groups<br><br>Sperm motility<br>*Competition* :<br>- lower in C vs R and TA (p < 0.05)<br>- lower in C vs themselves during the other two periods (p < 0.01)<br>*Resting period:*<br>- higher in C vs R (p < 0.05)<br><br>Note: absolute data were not mentioned, but depicted in graphics.	High intensity endurance cycling transiently decreases sperm motility, most likely due to testicular trauma and increased scrotal temperature.
Gebreegziabher et al.<br>(2004)<br>South Africa	Cross-sectional	Non-professional cyclists (C) (n=10)<br>(> 40 min/d, > 3 d/w)<br><br>Sedentary controls (SC) (n=10)	20-29	/	*Other assessments:*<br>- hormonal: no<br>- body composition: yes<br>- energy balance: no<br><br>*Sperm analysis criteria:*<br>WHO 4th edition<br><br>*Number of semen samples:*<br>Unspecified	Sperm volume (p > 0.05):<br>- C: 3.0 (2.1-3.4) ml<br>- SC: 2.3 (1.5-3.0) ml<br><br>Sperm count (p > 0.05):<br>- C: 38.2 (18.3-65.3) x 10^6/ml<br>- SC: 39.6 (26.7-75.4) x 10^6/ml<br><br>Total sperm count (p > 0.05):<br>- C: 86.6 (47.1-173.4) x 10^6<br>- SC: 90.4 (53.1-161.1) x 10^6<br><br>Sperm motility (p > 0.05):<br>*At time 0*<br>- C: 55 (50-78) %<br>- SC: 70 (55-80) %<br>*After 2 hours*<br>- C: 45 (31.6-70) %<br>- SC: 60 (50-70) %<br>*After 4 hours*<br>- C: 40 (21.0-50) %<br>- SC: 30 (26-58) %<br><br>Sperm morphology<br>*Normal* (p < 0.01):<br>- C: 19.5 (18.3-30.8) %<br>- SC: 41.5 (34.8-55.3) %<br>*Tapered* (p < 0.01):<br>- C: 22.5 (13.3-35.3) %<br>- SC: 4.5 (3.3-6.0) %<br>*Small acrosome* (p > 0.05):<br>- C: 15.0 (9.0-19.8) %<br>- SC: 17.5 (8.0-22.0) %<br>*Double head* (p > 0.05):<br>- C: 0 (0-0) %<br>- SC: 1.0 (0.0-1.8) %<br>*Immature forms* (p > 0.05):<br>- C: 1.0 (1.0-5.5) %<br>- SC: 2.5 (0.3-4.8) %<br>*Other forms* (p > 0.05):<br>- C: 0 (0-0) %<br>- SC: 0.0 (0.0-0.8) %<br><br>Sperm viability (p > 0.05):<br>- C: 62.0 (56.3-79.3) %<br>- SC: 76.0 (65.5-81.5) %	Endurance cycling is associated with a subclinical decrease of normal sperm morphology.

TT: total testosterone; FT: free testosterone; FSH: follicle stimulating hormone; LH: luteinizing hormone; C: cholesterol;

Table S4. Characteristics of literature found on triathletes.

Author, year, country	Study design	Participants	Age (years)	Intervention	Methods	Outcomes	Summary of results
Lucía et al. (1996) Spain	Longitudinal & cross-sectional	Professional cyclists (C) (n=12)<br>(884.46 ± 44.7 km/w)<br><br>Elite triathletes (TA) (n=9)<br>- running: 54.36 ± 7.2 km/w<br>- swimming: 14.36 ± 5.8 km/w<br>- cycling: 316.16 ± 79.0 km/w<br><br>Marathon runners (R) (n=10)<br>(94.26 ± 27.1 km/w)<br><br>Sedentary controls (SC) (n=9)	22-38	Follow-up during one sports season:<br>- precompetition<br>- competition<br>- resting period (2 weeks)	*Other assessments:*<br>- hormonal: yes<br>TT, FT, FSH, LH, C<br>- body composition: yes<br>- energy balance: no<br><br>*Sperm analysis criteria:*<br>WHO 3rd edition<br><br>*Number of semen samples:*<br>- 1 in pre-competition<br>- 1 in competition<br>- 1 after resting period	Semen morphology, volume and density ($p > 0.05$):<br>- no differences between or within groups<br><br>Sperm motility<br>*Competition* :<br>- lower in C vs R and TA ($p < 0.05$)<br>- lower in C vs themselves during the other two periods ($p < 0.01$)<br>*Resting period:*<br>- higher in C vs R ($p < 0.05$)<br><br>Note: absolute data were not mentioned, but depicted in graphics.	Triathlon does not negatively affect sperm quality.
Vaamonde et al. (2009) Spain	Cross-sectional	Physically active (PA) (n=16)<br>(> 1h, 3x/w)<br>(non-professional basketball, soccer, tennis, paddle ball)<br><br>Water polo players (WP) (n=14)<br>(1.5h, 5x/w)<br><br>Elite triathletes (TA) (n=15)<br>- running: 49.4 ± 7.4 km/w<br>- swimming: 11.3 ± 3.0 km/w<br>- cycling: 330.8 ± 56.0 km/w	17-38	/	*Other assessments:*<br>- hormonal: no<br>- body composition: yes<br>- energy balance: no<br><br>*Sperm analysis criteria:*<br>- WHO 4th edition<br>- Kruger's strict criteria (1995) for morphology<br><br>*Number of semen samples:*<br>Unspecified	Sperm volume ($p = 0.48$):<br>- PA: 3.2 ± 0.9 ml<br>- WP: 3.4 ± 1.3 ml<br>- TA: 2.9 ± 0.9 ml<br><br>Sperm concentration ($p = 0.04$):<br>- PA: 61.0 ± 23.0 x 10^6/mL<br>- WP: 58.0 ± 24.4 x 10^6/mL<br>- TA: 48.2 ± 14.7 x 10^6/mL (lower vs PA and WP)<br><br>Total sperm number ($p = 0.03$):<br>- PA: 191.8 ± 73.4 x 10^6<br>- WP: 196.6 ± 85.4 x 10^6<br>- TA: 141.3 ± 58.0 x 10^6 (lower vs PA and WP)*<br>* 3 oligospermic subjects<br><br>Sperm morphology<br>*Normal forms* ($p = 0.01$):<br>- PA: 15.2 ± 1.2 %<br>- WP: 9.7 ± 3.0 %<br>- TA: 4.7 ± 2.2 % (lower vs PA and WP)<br><br>Sperm motility[†]<br>*Type "a"* ($p = 0.03$):<br>- PA: 31.1 ± 9.7 %<br>- WP: 23.6 ± 8.8 %<br>- TA: 31.4 ± 8.7 % (higher vs WP)<br>*Type "b"* ($p = 0.02$):<br>- PA: 25.6 ± 9.1 %<br>- WP: 28.8 ± 12.3 %<br>- TA: 18.9 ± 7.6 % (lower vs PA and WP)<br>*Type "a+b"* ($p = 0.34$):<br>- PA: 56.7 ± 6.5 %<br>- WP: 52.5 ± 11.1 %<br>- TA: 50.3 ± 8.9 %<br>*Type "c"* ($p = 0.41$):<br>- PA: 10.4 ± 5.0 %<br>- WP: 14.3 ± 6.6 %<br>- TA: 11.9 ± 6.3 %<br>*Type "d"* ($p = 0.03$):<br>- PA: 33.0 ± 7.1 %<br>- WP: 33.3 ± 11.3 %<br>- TA: 38.4 ± 7.2 % (higher vs PA)	Triathlon negatively affects multiple sperm parameters. The amount of normal forms is clinically decreased and may therefore lead to fertility problems.
Vaamonde et al. (2009) Spain	Cross-sectional	Elite triathletes (n=15)<br>- running: 49.42 ± 7.37 km/w<br>- swimming: 11.31 ± 3.05 km/w<br>- cycling: 330.77 ± 56.04 km/w	29-38	/	*Other assessments:*<br>- hormonal: no<br>- body composition: yes<br>- energy balance: no<br><br>*Sperm analysis criteria:*<br>Kruger's strict criteria (1995) for morphology<br><br>*Number of semen samples:*<br>Unspecified	Sperm morphology correlation:<br>- total weekly volume: $r = -0.29$ ($p > 0.05$)<br>- cycling volume: $r = -0.71$ ($p < 0.05$)<br>- running volume: $r = -0.12$ ($p > 0.05$)<br>- swimming volume: $r = 0.09$ ($p > 0.05$)	Sperm morphology (normal forms) and weekly cycling volume are inversely correlated. Cycling more than 300 km/w may impair fertility potential.
Vaamonde et al. (2018) Spain	Cross-sectional	Elite triathletes (n=12)<br>- running: ± 2.600 km/y<br>- swimming: ± 416 km/y<br>- cycling: ± 13.000 km/y	24-30	/	*Other assessments:*<br>- hormonal: yes<br>TT, C<br>- body composition: no<br>- energy balance: no<br><br>*Sperm analysis criteria:*<br>WHO 5th edition<br><br>*Number of semen samples:*<br>Unspecified	Sperm volume ($p > 0.05$):<br>3.3 ± 1.7 ml<br><br>Sperm concentration ($p > 0.05$):<br>50.4 ± 46.7 x 10^6/ml<br><br>Total sperm number ($p > 0.05$):<br>175.8 ± 150.6 x 10^6<br><br>Sperm morphology<br>*Normal forms* ($p > 0.05$):<br>5.3 ± 2.7 %<br><br>DNA fragmentation ($p < 0.05$):<br>20.4 ± 6.1% (elevated)<br><br>Sperm motility ($p > 0.05$):<br>*Total motility*<br>58.8 ± 12.5 %<br>*Progressive motility*<br>50.8 ± 16.3 %<br>*Non-progressive motility*<br>7.6 ± 4.7 %<br><br>Round cells ($p < 0.05$):<br>*Number*<br>2.8 ± 1.5 x 10^6/ml (elevated)<br>*Positive correlation*<br>- progressive sperm motility: $r = 0.77$ ($p = 0.01$)<br>- sperm morphology: $r = 0.72$ ($p = 0.02$)<br>*Negative correlation*<br>- DNA fragmentation: $r = -0.67$ ($p = 0.04$)	Triathlon may negatively affect fertility potential because DNA fragmentation and round cells are clinically increased.

TT: total testosterone; FT: free testosterone; FSH: follicle stimulating hormone; LH: luteinizing hormone; C: cholesterol;

[†] Sperm motility can be classified in different types. Type "a" are spermatozoa moving at > 20 mm/s, type "b" at 5-20 mm/s, type "c" at < 5 mm/s and type "d" are static.

Table S5: Assessment of methodological quality and potential risk of bias using the JBI Critical Appraisal Tool.

JBI checklist questions	Arce et al.	Ayers et al.	Bagatell et al.	De Souza et al.	Gebreegziabher et al.	Griffith et al.	Hall et al.	Jensen et al.	Lucìa et al.	Roberts et al.	Vaamonde et al.	Vaamonde et al.	Vaamonde et al.
1. Were the criteria for inclusion in the sample clearly defined?	Y	N	Y	Y	Y	N	Y	N	Y	N	Y	Y	Y
2. Were the study subjects and the setting described in detail?	Y	Y	Y	Y	Y	N	Y	N	N	N	Y	Y	Y
3. Was the exposure measured in a valid and reliable way?	Y	Y	Y	Y	N	N	Y	Y	Y	N	Y	Y	N
4. Were objective, standard criteria used for measurement of the condition?	Y	Y	Y	Y	Y	Y	Y	Y	Y	Y	Y	Y	Y
5. Were confounding factors identified?	N	N	N	N	N	N	N	N	N	N	N	N	N
6. Were strategies to deal with confounding factors stated?	N/A	N/A	N/A	N/A	N/A	N/A	N/A	N/A	N/A	N/A	N/A	N/A	N/A
7. Were the outcomes measured in a valid and reliable way?	Y	N	Y	Y	Y	Y	Y	Y	Y	Y	Y	Y	Y
8. Was appropriate statistical analysis used?	Y	Y	Y	Y	Y	Y	Y	Y	Y	Y	Y	Y	Y

Table S6: Variation in semen analysis guidelines.

Guideline (year of publication)	Nomenclature	Semen volume (ml)	Sperm concentration (10^6/ml)	Total sperm count (10^6/ejaculate)	Total sperm motility (%)	Progressive sperm motility (%)	Sperm morphology (% normal form)
**Bremner et al.† (1981)**							
**Kruger's strict criteria (1986)**	Below threshold						> 14
**WHO 2nd edition (1987)**	Normal values	≥ 2	≥ 20	≥ 40	≥ 50	≥ 25	≥ 50
**WHO 3rd edition (1992)**	Normal values	≥ 2	≥ 20	≥ 40	≥ 50	≥ 25	≥ 50
**Kruger's strict criteria (1995)**	Below threshold		≥ 2				4
**WHO 4th edition (1999)**	Reference values		≥ 20	≥ 40	≥ 50	≥ 25	≥ 15
**WHO 5th edition (2010)**	Reference values of fertile man (5th centile lower reference limits)	1.5	15	39	40	32	4
**WHO 6th edition (2021)**	Distribution of semen variables from fertile men (5th centile)	1.4	16	39	42	30	4

† No reference values available

APPENDIX S1: PRISMA 2020 Statement

Section and Topic	Item #	Checklist item	Page where item is reported
**TITLE**			
Title	1	Identify the report as a systematic review.	1
**ABSTRACT**			
Abstract	2	See the PRISMA 2020 for Abstracts checklist.	3
**INTRODUCTION**			
Rationale	3	Describe the rationale for the review in the context of existing knowledge.	5-6
Objectives	4	Provide an explicit statement of the objective(s) or question(s) the review addresses.	6
**METHODS**			
Eligibility criteria	5	Specify the inclusion and exclusion criteria for the review and how studies were grouped for the syntheses.	7
Information sources	6	Specify all databases, registers, websites, organisations, reference lists and other sources searched or consulted to identify studies. Specify the date when each source was last searched or consulted.	7
Search strategy	7	Present the full search strategies for all databases, registers and websites, including any filters and limits used.	Suppl data
Selection process	8	Specify the methods used to decide whether a study met the inclusion criteria of the review, including how many reviewers screened each record and each report retrieved, whether they worked independently, and if applicable, details of automation tools used in the process.	8
Data collection process	9	Specify the methods used to collect data from reports, including how many reviewers collected data from each report, whether they worked independently, any processes for obtaining or confirming data from study investigators, and if applicable, details of automation tools used in the process.	8
Data items	10a	List and define all outcomes for which data were sought. Specify whether all results that were compatible with each outcome domain in each study were sought (e.g. for all measures, time points, analyses), and if not, the methods used to decide which results to collect.	8
	10b	List and define all other variables for which data were sought (e.g. participant and intervention characteristics, funding sources). Describe any assumptions made about any missing or unclear information.	8
Study risk of bias assessment	11	Specify the methods used to assess risk of bias in the included studies, including details of the tool(s) used, how many reviewers assessed each study and whether they worked independently, and if applicable, details of automation tools used in the process.	8-9
Effect measures	12	Specify for each outcome the effect measure(s) (e.g. risk ratio, mean difference) used in the synthesis or presentation of results.	8
Synthesis methods	13a	Describe the processes used to decide which studies were eligible for each synthesis (e.g. tabulating the study intervention characteristics and comparing against the planned groups for each synthesis (item #5)).	8-9
	13b	Describe any methods required to prepare the data for presentation or synthesis, such as handling of missing summary statistics, or data conversions.	NA
	13c	Describe any methods used to tabulate or visually display results of individual studies and syntheses.	8-9
	13d	Describe any methods used to synthesize results and provide a rationale for the choice(s). If meta-analysis was performed, describe the model(s), method(s) to identify the presence and extent of statistical heterogeneity, and software package(s) used.	NA
	13e	Describe any methods used to explore possible causes of heterogeneity among study results (e.g. subgroup analysis, meta-regression).	NA
	13f	Describe any sensitivity analyses conducted to assess robustness of the synthesized results.	NA
Reporting bias	14	Describe any methods used to assess risk of bias due to missing results in a synthesis (arising from reporting biases).	NA

Section and Topic	Item #	Checklist item	Page where item is reported
assessment			
Certainty assessment	15	Describe any methods used to assess certainty (or confidence) in the body of evidence for an outcome.	NA
**RESULTS**			
Study selection	16a	Describe the results of the search and selection process, from the number of records identified in the search to the number of studies included in the review, ideally using a flow diagram.	Fig 1
	16b	Cite studies that might appear to meet the inclusion criteria, but which were excluded, and explain why they were excluded.	NA
Study characteristics	17	Cite each included study and present its characteristics.	10-14
Risk of bias in studies	18	Present assessments of risk of bias for each included study.	Suppl material
Results of individual studies	19	For all outcomes, present, for each study: (a) summary statistics for each group (where appropriate) and (b) an effect estimate and its precision (e.g. confidence/credible interval), ideally using structured tables or plots.	Tables
Results of syntheses	20a	For each synthesis, briefly summarise the characteristics and risk of bias among contributing studies.	10-14
	20b	Present results of all statistical syntheses conducted. If meta-analysis was done, present for each the summary estimate and its precision (e.g. confidence/credible interval) and measures of statistical heterogeneity. If comparing groups, describe the direction of the effect.	NA
	20c	Present results of all investigations of possible causes of heterogeneity among study results.	NA
	20d	Present results of all sensitivity analyses conducted to assess the robustness of the synthesized results.	NA
Reporting biases	21	Present assessments of risk of bias due to missing results (arising from reporting biases) for each synthesis assessed.	NA
Certainty of evidence	22	Present assessments of certainty (or confidence) in the body of evidence for each outcome assessed.	NA
**DISCUSSION**			
Discussion	23a	Provide a general interpretation of the results in the context of other evidence.	14-16
	23b	Discuss any limitations of the evidence included in the review.	16-17
	23c	Discuss any limitations of the review processes used.	16-17
	23d	Discuss implications of the results for practice, policy, and future research.	17
**OTHER INFORMATION**			
Registration and protocol	24a	Provide registration information for the review, including register name and registration number, or state that the review was not registered.	7
	24b	Indicate where the review protocol can be accessed, or state that a protocol was not prepared.	7
	24c	Describe and explain any amendments to information provided at registration or in the protocol.	NA
Support	25	Describe sources of financial or non-financial support for the review, and the role of the funders or sponsors in the review.	18
Competing interests	26	Declare any competing interests of review authors.	18
Availability of data, code and other materials	27	Report which of the following are publicly available and where they can be found: template data collection forms; data extracted from included studies; data used for all analyses; analytic code; any other materials used in the review.	NA
